# Inside a Dual Secretory Cell: Ultrastructural Insights into Mucilage and Phenolic Secretion in *Mimosa* Species (Leguminosae)

**DOI:** 10.3390/plants15111592

**Published:** 2026-05-22

**Authors:** Thaís Alves De Sousa, Thais Cury De Barros, Leonardo Maurici Borges, Simone Pádua Teixeira

**Affiliations:** 1 Programa de Pós-Graduação em Biologia Comparada, Faculdade de Filosofia, Ciências e Letras de Ribeirão Preto, Universidade de São Paulo, Ribeirão Preto 14040-130, SP, Brazil; sousathais@usp.br; 2 Departamento de Ciências Farmacêuticas, Faculdade de Ciências Farmacêuticas de Ribeirão Preto, Universidade de São Paulo, Ribeirão Preto 14040-903, SP, Brazil; 3 Departamento de Biodiversidade e Bioestatística, Instituto de Biociências, Universidade Estadual Paulista “Júlio de Mesquita Filho”, Botucatu 18618-689, SP, Brazil; tc.barros@unesp.br; 4 Departamento de Botânica, Universidade Federal de São Carlos, São Carlos 13565-905, SP, Brazil; aquitemcaqui@gmail.com

**Keywords:** anatomy, Fabaceae, gland, microscopy, secondary metabolites, secretory idioblast

## Abstract

The co-occurrence of mucilage and phenolic compounds within the same secretory cell is rarely documented in plants. Recently, such cells were reported in vegetative and floral organs of sensitive legumes (*Mimosa*), but without detailed subcellular analysis. To address this gap, we used transmission electron microscopy to examine the organelles involved in biosynthesis, the intracellular sites of metabolite storage, and the secretion processes across floral and foliar organs in five *Mimosa* species. Secretory epidermal cells of sepals, petals, and leaf blades produce both mucilage and phenolics, with no significant differences between organ types. Dictyosomes, rough endoplasmic reticulum, and plastids predominated in the cytoplasm of the secretory cell during biosynthesis. Dictyosomes may mediate mucilage production, the rough endoplasmic reticulum may be involved in phenolic synthesis, and plastids may contribute to the biosynthesis of both compounds. These metabolites are stored in distinct cellular domains: phenolics accumulate in a large vacuole near the outer periclinal wall, while mucilage is deposited between the microfibrils of the inner periclinal wall. This spatial separation is evident by the distention of the inner periclinal wall due to mucilage accumulation. The absence of karyokinesis and phragmoplast formation during metabolite segregation confirms that these secretory cells have two different functional domains, forming a uniseriate rather than biseriate epidermis. Notably, the inclusion of several species in the ultrastructural analyses enhances the significance of these findings.

## 1. Introduction

A secretory cell is specialized in producing and storing secondary metabolites, which may or may not be released. These compounds are crucial for plant survival in diverse environments, helping to mitigate stress from solar radiation, water scarcity or excess, herbivore and pathogen attacks, and to attract pollinators and dispersers [1,2]. Mucilage and phenolics are among the compounds produced by secretory cells, which have been recorded in floral and foliar organs of various angiosperm families such as Asteraceae [3], Cannabaceae [4,5], Leguminosae [6,7,8,9], Malvaceae [10], Primulaceae [11], Orchidaceae [12], Rhamnaceae [4,13], Ulmaceae and Urticaceae [4].

Mucilage is a hydrophilic substance that consists of high-molecular-weight acidic polysaccharides such as arabinogalactans, rhamnogalacturonans, and other pectic and hemicellulosic fractions [14]. It is synthesized in the endoplasmic reticulum and Golgi complex, where it is packaged in vesicles that can be directed to vacuoles or secreted into the periplasmic space [1,2,15]. Phenolic compounds are a class of secondary metabolites characterized by aromatic rings containing hydroxyl groups, mainly derived from the phenylpropanoid pathway [16,17,18,19]. Examples include flavonoids, tannins, lignans, and phenolic acids. They are synthesized from enzymes located in the cytoplasm and plastids, being stored in vacuoles, but they can also accumulate in the cell wall or cytoplasm [17,20]. While mucilage cells have been the subject of ultrastructural studies [3,4,10,21], phenolic cells are less frequently studied in detail [5,8,13], hampering our knowledge on the diversity and function of such secretory cells.

The localization of mucilage cells in the plant body, the cellular mechanisms of mucilage production, and their storage sites vary greatly [6,22], particularly between phylogenetically distant groups. In Orchidaceae, Cactaceae, and Rhamnaceae, studies to date indicate that these mechanisms are distinct: in Orchidaceae, it is produced in the cytoplasm and deposited in the cell wall [12]; in Cactaceae, via the hydrolysis of primary cell walls that serve a mechanical role during bud growth [23]; and in Rhamnaceae, by contiguous mucilaginous cells forming an extensive secretory system through cell wall dissolution, with mucilage synthesized in the cytoplasm and stored in both vacuoles and the periplasmic space [4]. A mucilage secretory system similar to that of Rhamnaceae occurs in the leaves of *Hibiscus schizopetalus* (Mast.) Hook.f. (Malvaceae) [12] and *Araucaria angustifolia* (Bertol.) Kuntze (Araucariaceae) [21], as well as in the ovule integument of *Hieracium* L. and *Pilosella* Vaill. (Asteraceae) [3]. All these studies indicate that mucilage plays an important role in water storage and/or as a carbohydrate reserve.

Similarly to mucilage cells, phenolic cells exhibit variable sizes and shapes and even different forms of storage, with compounds being stored in a large central vacuole or in tannosomes [8,17]. Recent studies show that phenolic compounds can influence the function of other secretory structures. Examples include the internal phenolic layer that helps contain nectar produced in foliar nectaries of *Colubrina glandulosa* Perkins (Rhamnaceae), and the phenolic epithelium of secretory ducts found in *Rhamnidium elaeocarpum* Reissek (Rhamnaceae) [13]. In *Humulus lupulus* L. (Cannabaceae), layers of phenolic cells found near laticifers throughout the plant body are characterized by cytoplasm rich in amyloplasts, rough endoplasmic reticulum, mitochondria, and plastids with atypical morphology [5]. Phenolic compounds have been reported to serve multiple roles, including defense against herbivores, antimicrobial and antioxidant activity against pathogens [17,24], and protection from ultraviolet radiation through absorption [25].

From the above, it is clear that mucilage and phenolics are widespread across different angiosperm groups. However, the occurrence of both compounds within a single secretory cell remains sparsely documented. In legumes, despite the high species richness of the family [26], available evidence is based on a limited number of studies and species [27,28], including more recent records in the sensitive plants of the genus *Mimosa* [27,29]. *Mimosa* is a member of subfamily Caesalpinioideae, tribe Mimoseae, and is characterized by bipinnate leaves, inflorescences in glomerules or spikes with pink or light-yellow staminal filaments, and craspedial fruits (dry, indehiscent fruit that is segmented transversely into articles) [30,31,32,33]. The genus includes around 600 species distributed in a wide range of environments mostly across the Neotropics, but also reaching Africa and Asia [31].

Here we aim to understand the organization of the subcellular machinery involved in the synthesis and storage of mucilage and phenolics in the same single secretory cell. To achieve this, we study the ultrastructure (via transmission electron microscopy) of foliar and floral organs of five *Mimosa* species previously known to bear these atypical cells to answer the following questions: (i) Which organelles are involved in the simultaneous synthesis of mucilage and phenolic compounds? (ii) Where are these metabolites stored in the cell? (iii) Are the secretion processes of these metabolites similar in the organs and species analyzed?

## 2. Results

**Figure 1:**
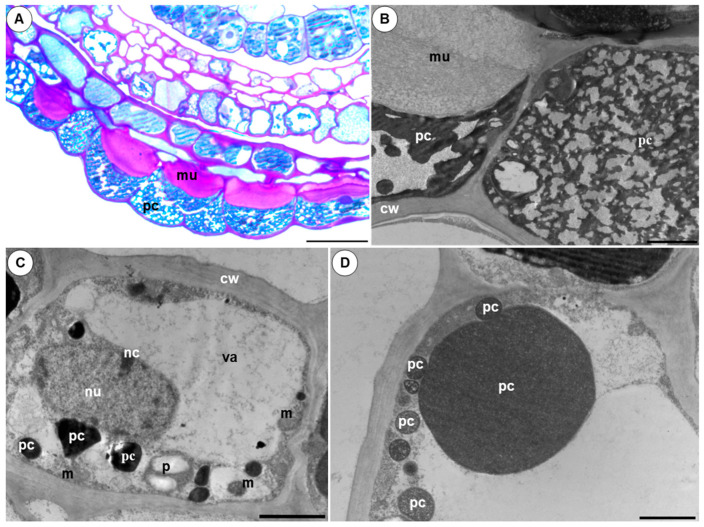
Photomicrographs (**A**) and electron micrographs (**B**–**D**) of the mucilage and phenolic-secreting cells in the sepal of *Mimosa caesalpiniifolia*. (**A**): Mucilage (in pink) and phenolic compounds (in blue-green) in the sepal epidermis. (**B**): Detail of the cell with two domains, one containing mucilage between the microfibrils of the inner periclinal wall and another containing a large phenolic vacuole. Note the contiguous phenolic cells. (**C**): Cell with a prominent and peripheral nucleus, a central vacuole, small peripheral phenolic vacuoles, mitochondria, and nearby amyloplasts. (**D**): Cell containing vacuoles of varying sizes filled with phenolic compounds showing a striated appearance. Symbols: cw = cell wall; m = mitochondria; mu = mucilage; nu = nucleus; nc = nucleolus; p = plastid; pc = phenolic compound; va = vacuole. Scales: 20 μm (**A**); 2 μm (**B**–**D**).

**Figure 2:**
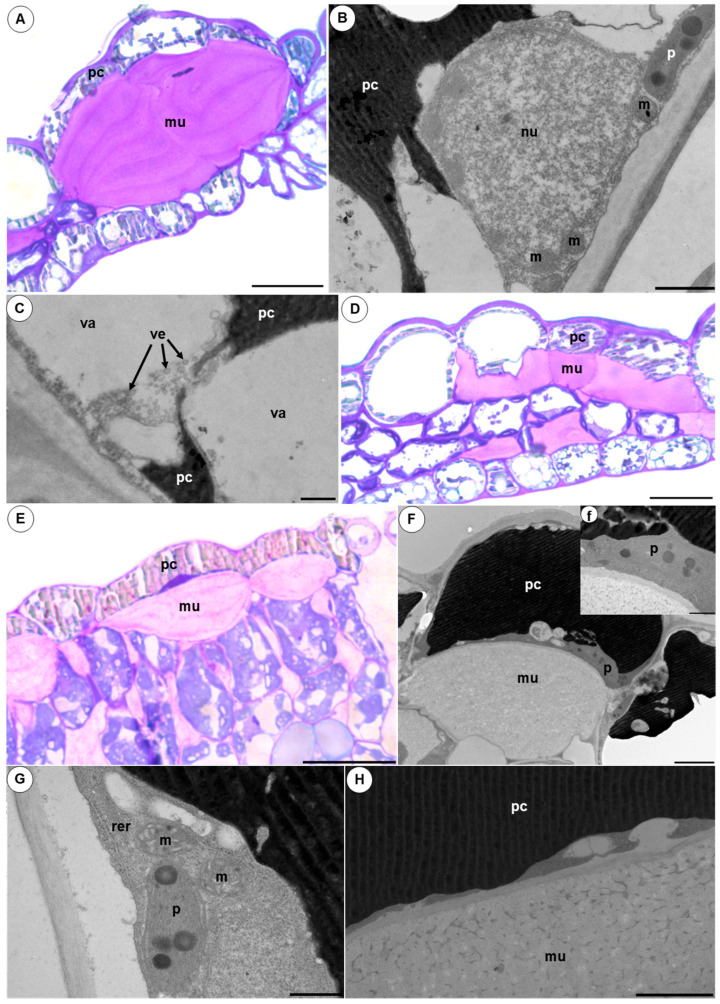
Photomicrographs (**A**,**D**,**E**) and electron micrographs (**B**,**C**,**F**–**H**) of the mucilage and phenolic-secreting cells in the petal (**A**–**D**) and leaflet blade (**E**–**H**) of *Mimosa diplotricha*. (**A**): Mucilage (in pink) in the petal epidermis. (**B**): Cell with a prominent and peripheral nucleus, with nearby mitochondria and plastid. (**C**): Cell with small vesicles (arrows) near the tonoplast of the phenolic vacuole. (**D**): Contiguous cells with ruptured walls. (**E**): Mucilage (in pink) and phenolic compounds (in blue-green) in the leaflet blade epidermis. (**F**): Cell with two domains: phenolic vacuole facing the outer periclinal wall and mucilage between the microfibrils of the inner periclinal wall. (**f**): Magnified view of F showing a plastid with osmiophilic inclusions in detail. (**G**): Peripheral portion of the cytoplasm with rough endoplasmic reticulum, mitochondria, and plastid with osmiophilic inclusions. (**H**): Detail of the dual-domain cell. Note the striated appearance of the phenolics in G and H. Symbols: m = mitochondria; mu = mucilage; nu = nucleus; p = plastid; pc = phenolic compound; rer = rough endoplasmic reticulum; va = vacuole; ve = vesicle. Scale bars: 20 μm (**A**,**D**,**E**); 2 μm (**F**); 1 μm (**B**,**C**,**H**); 500 nm (**G**,**f**).

**Figure 3:**
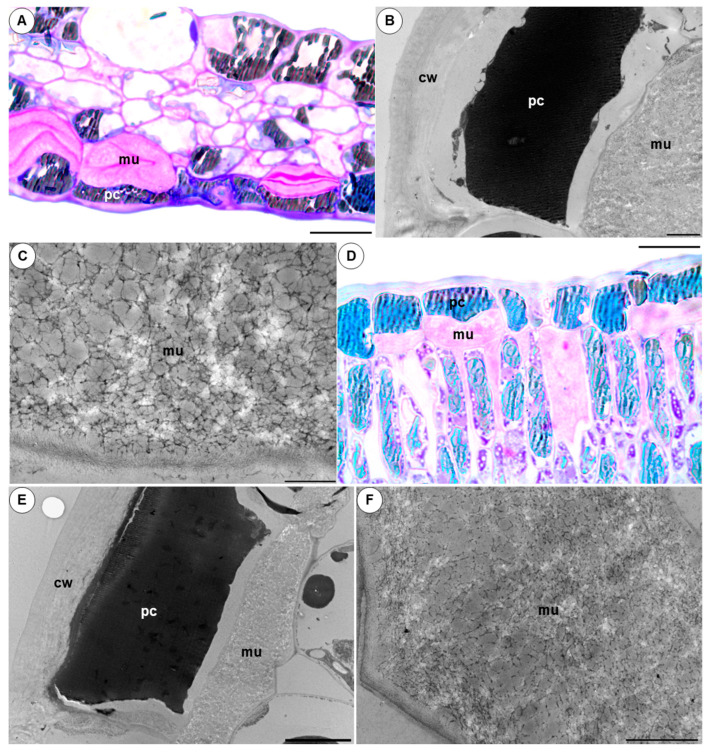
Photomicrographs (**A**,**D**) and electron micrographs (**B**,**C**,**E**,**F**) of the mucilage and phenolic-secreting cells in the petal (**A**–**C**) and leaf blade (**D**–**F**) of *Mimosa myrioglandulosa*. (**A**): Mucilage (in pink) and phenolic compounds (in blue-green) in the petal epidermis. (**B**): Cell with two domains: phenolic vacuole facing the outer periclinal wall and mucilage between the microfibrils of the inner periclinal wall. Note the striated appearance of the phenolics. (**C**): Detail showing the mucilage located on the inner periclinal wall. (**D**): Cells with ruptured internal periclinal walls. Note the extrusion of mucilage into the intercellular spaces. (**E**): Cell with two domains: phenolic vacuole facing the outer periclinal wall and mucilage between the microfibrils of the inner periclinal wall. (**F**): Detail showing the mucilage located on the inner periclinal wall. Symbols: cw = cell wall; mu = mucilage; pc = phenolic compounds. Scale bars: 100 μm (**A**,**D**); 5 μm (**E**); 2 μm (**B**); 1 μm (**F**); 500 nm (**C**).

**Figure 4:**
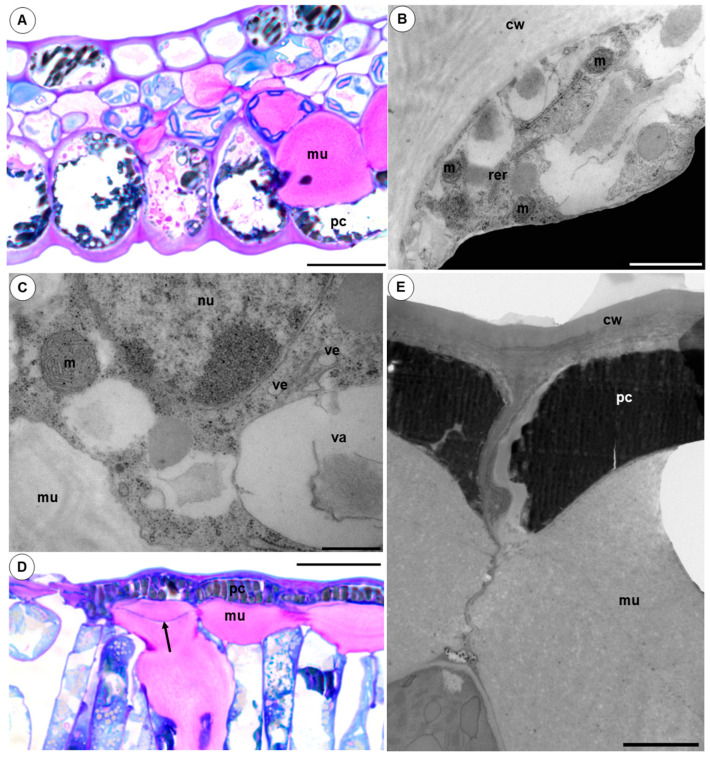
Photomicrographs (**A**,**D**) and electron micrographs (**B**,**C**,**E**) of the mucilage and phenolic-secreting cells in the petal (**A**–**C**) and leaf blade (**D**–**E**) of *Mimosa paludosa*. (**A**): Panoramic view of petal tissues showing mucilage (pink) and phenolic compounds (blue-green) in epidermal secretory cells. (**B**): Cell with thick walls and peripheral portion of the cytoplasm containing mitochondria and rough endoplasmic reticulum. (**C**): Cell with nucleus, mitochondria, vacuole, and vesicles. (**D**): Panoramic view of leaflet blade epidermal cells showing mucilage (pink) and phenolic compounds (blue-green). Note the inner portion of the cell wall (arrow). (**E**): Detail of the dual-domain cells. Note the striated appearance of the phenolics. Symbols: cw = cell wall; m = mitochondria; mu = mucilage; nu = nucleus; pc = phenolic compound; va = vacuole; ve = vesicle; rer = rough endoplasmic reticulum. Scale bars: 100 μm (**A**,**D**); 2 μm (**E**); 1 μm (**B**); 500 nm (**C**).

**Figure 5:**
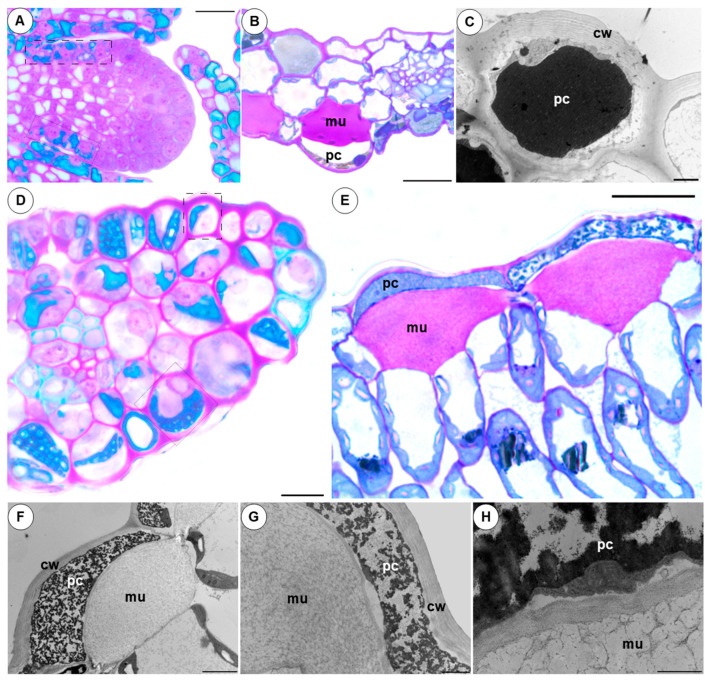
Photomicrographs (**A**,**B**,**D**,**E**) and electron micrographs (**C**,**F**–**H**) of the mucilage and phenolic-secreting cells of *Mimosa pudica*. (**A**–**C**). Differentiating ((**A**)—dotted rectangle) and differentiated (**B**–**C**) dual-domain secretory cell (phenolic and mucilage) in the petal epidermis. Note the thick outer periclinal wall and large phenolic vacuole in (**C**). (**D**–**H**): Differentiating ((**D**)—dotted rectangle) and differentiated (**E**–**H**) dual-domain secretory cell (phenolic and mucilage) in the leaf blade epidermis. Note the mucilage between the loose microfibrils in the inner periclinal wall in (**G**,**H**). Symbols: cw = cell wall; mu = mucilage; pc = phenolic compound. Scale bars: 100 μm (**A**,**D**); 20 μm (**C**,**H**); 5 μm (**F**); 2 μm (**B**,**E**); 500 nm (**G**).

*Mimosa caesalpiniifolia* Benth., *M. diplotricha* C. Wright ex Sauvalle, *M. myrioglandulosa* V. F. Dutra & F. C. P. Garcia, *M. paludosa* Benth. and *M. pudica* L. exhibit cells that simultaneously secrete both mucilage and phenolic compounds (Figure 1, Figure 2, Figure 3, Figure 4 and Figure 5). These cells are located in the epidermis of the leaf blade, sepals, and/or petals (Table 1) and possess thick periclinal walls (Figure 1B, Figure 2F, Figure 3B,E, Figure 4B,E and Figure 5B,E,F).

Mucilage and phenolic compounds are stored in different domains at opposite sides of the cell. Mucilage accumulates between the microfibrils of the inner periclinal wall while phenolics are stored in a larger vacuole facing the outer periclinal wall (Figure 1A,B, Figure 2A,E,F, Figure 3A–F, Figure 4A,D,E and Figure 5A,D–F). This spatial separation is defined by the innermost portion of the internal periclinal wall, which is distended by mucilage accumulation (Figure 4D), without differentiated electron density or granular organization (Figure 4E).

In the early stages of secretory process, secretory cells exhibit a prominent peripheral nucleus, as well as peripheral organelles such as rough endoplasmic reticulum, mitochondria, plastids with osmophilic inclusions, and vesicles (Figure 1C, Figure 2B,C,G, Figure 4B,C and Figure 5G). They also contain a large central vacuole and small peripheral vacuoles filled with phenolic compounds (Figure 1C,D). These smaller vacuoles gradually merge with the central vacuole (Figure 1D) on the side of the cell opposite the mucilage-storing wall (Figure 1B and Figure 5G). Phenolic compounds are synthesized before mucilage and are stored in vacuoles through centripetal accumulation, from the cell periphery toward the center (Figure 5C,H), while exhibiting a striated appearance (Figure 1B,D, Figure 2B,G,H, Figure 3B,E and Figure 4E).

In the final stages of secretory process, the periclinal and/or anticlinal walls of contiguous cells may rupture, leading to mucilage leakage into the intercellular spaces, as observed in *M. diplotricha* (Figure 2D), *M. myrioglandulosa* (Figure 3D), and *M. paludosa* (Figure 4D). No evident rupture of the vacuole containing phenolic compounds was observed.

## 3. Discussion

The concurrent production of mucilage and phenolic compounds within the same secretory cell has rarely been documented in the literature from either structural or ultrastructural perspectives. Previous studies have suggested that phenolic compounds may act in association with other chemical constituents, including mucilage, contributing to plant defense and protection [5,13,34,35].

### 3.1. Are Mucilage and Phenolics Secreted in Dual Functional-Domains or in Contiguous Cells?

In all five species studied, mucilage and phenolic compounds are produced by secretory epidermal cells in all organs examined (sepals, petals, and leaf blades). In these cells, phenolics accumulate in a vacuole adjacent to the outer periclinal wall, while mucilage builds up within the inner periclinal wall, which undergoes a process of “mucilagination” or “gelatinization,” as described by Bredenkamp and Van Wyk [36] for *Passerina* L. (Thymelaeaceae). The resulting mucilage accumulation distends the inner periclinal wall, leading to the separation of its innermost portion. This pattern of mucilage deposition contrasts with that found in cells where mucilage accumulates between the plasmalemma and the cell wall [36]. Such cellular organization raises questions about the nature of the epidermis in these species: Does it represent a biseriate epidermis formed by cell division, or is it a uniseriate epidermis with individual cells exhibiting dual functional domains?

Since we observed no evidence of karyokinesis or phragmoplast formation during metabolite segregation, we infer that the secretory cells do not divide after synthesizing mucilage and phenolics. Thus, the epidermis cannot be classified as biseriate, despite its appearance. This contrasts with the pattern described in *Acosmium cardenasii* H.S.Irwin & Arroyo (Leguminosae), which presents clear karyokinesis and phragmoplast formation during the development of a true biseriate epidermis [37].

Therefore, the most plausible interpretation is that the secretory cells in *Mimosa* are part of a uniseriate epidermis that exhibits both functional and structural domains. This pattern resembles that observed in the secretory cells of *Cuphea calophylla* Cham. & Schltdl. (Lythraceae) [38], *Muntingia calabura* L. (Muntingiaceae) [39], *Passerina* L. (Thymelaeaceae) [36], and even in the fibrotracheids of *Hypericum androsaemum* L. (Hypericaceae) [40].

When comparing the secretory cells of *Mimosa* to fibrotracheids, a key distinction lies in the fact that fibrotracheids undergo both karyokinesis and cytokinesis, producing two nuclei, one in each compartment, whereas no such nuclear division was observed in *Mimosa*. In contrast to the secretory cells of *Myrsine umbellata* [11] and *Cuphea calophylla* [38], where the formation of a new cell wall dividing the cell into two functional domains has been documented, no such wall was identified in *Mimosa*. Consequently, the secretory cells of *Mimosa* exhibit dual functional domains that coexist within a single undivided cell, representing an interesting mode of subcellular organization and spatial separation of metabolites.

### 3.2. Mucilage and Phenolic-Secreting Dynamics

No differences were detected in the secretion process of mucilage and phenolic compounds between floral and foliar organs in four of the five studied *Mimosa* species. The exception was *M. caesalpinifolia*, in which mucilage was not detected in the leaves [29], precluding this comparison.

On the other hand, the synthesis of these compounds can be associated with different sets of organelles found in the secretory cell. Mucilage synthesis is mediated by dictyosomes and plastids, whereas phenolic biosynthesis involves multiple organelles. The formation of phenolic precursors likely occurs in plastids [41], while the rough endoplasmic reticulum appears to participate in their subsequent modification and transport [8]. The role of dictyosomes on mucilage synthesis was to be expected based on similar reports for *Araucaria angustifolia* [21], *Cinnamomum burmanni* Bl. [42], *Entelea arborescens* R. Br. [43], *Euglena gracilis* Klebs [44], *Hibiscus schizopetalus* [10], *Hieracium alpinum* [3], *Pilosella officinarum* [3], *Opuntia polyacantha* Haw. [45], *Schizolobium parahyba* (Vell.) Blake [15], and *Tilia vulgaris* Hayne [43]. Similarly, extensive participation of the rough endoplasmic reticulum in phenolic compound synthesis has been registered for other legume species [8]. The presence of plastids in the secretory cells of the analyzed *Mimosa* species appears to be associated both with the enzymatic processes involved in mucilage storage, by facilitating the loosening of cell wall microfibrils [46], and with the synthesis of phenolic compounds in response to herbivory [47,48]. This interpretation is consistent with the striated appearance of the phenolic compounds accumulated within the vacuole, particularly evident in *M. caesalpiniifolia*, *M. diplotricha*, *M. myrioglandulosa*, and *M. paludosa*. Such a pattern may reflect the chemical nature of these compounds and suggest the presence of condensed tannins [8], which are commonly related to antioxidant activity and protection against herbivores and pathogens [20,25]. However, more refined chemical analyses are still required for more precise characterization.

The transport and storage of phenolic compounds and mucilage follow similar mechanisms, although they occur at different stages of the secretion process in the studied species of *Mimosa*. Phenolic compounds are synthesized earlier than mucilage and are stored in vacuoles that fill centripetally, from the cell periphery toward the center. Likewise, mucilage is transported in vesicles directed toward the cell wall. The polarization of cellular compartments containing either mucilage or phenolics becomes evident early during the accumulation phase of these compounds.

The storage of mucilage in the cell wall observed here for *Mimosa* species resembles that reported for species belonging to at least 88 other families [49,50]. In these families, mucilage is deposited in the cell wall or between the cell wall and the plasma membrane [3,4,10,12,22,23,42,45,50]. Storage of phenolic compounds in vacuoles is a common condition in angiosperms [8,19]. Small vacuoles with phenolics merge to form a single and large storage unit [8,51] and the phenolic storage occurs centripetally, i.e., from the tonoplast to the center of the vacuole [8,52].

Mucilage accumulates in large amounts in *Mimosa diplotricha*, *M. myrioglandulosa*, and *M. paludosa*, leading to rupture of the secretory cell wall and subsequent release into intercellular spaces. This process was consistently observed under both light and transmission electron microscopy, suggesting a relationship with increased intracellular pressure, as reported in other plant groups, including Araucariaceae [21], Asteraceae [3], Begoniaceae [53], Malvaceae [10], most families of Rosales [4], and other Leguminosae [7]. This pattern indicates that mucilage deployment is a highly dynamic mechanism and may play an active role in stress mitigation and defense signaling against herbivores, pathogens, solar radiation, and water loss in reproductive tissues [14,28,29,54].

## 4. Materials and Methods

**Figure 6:**
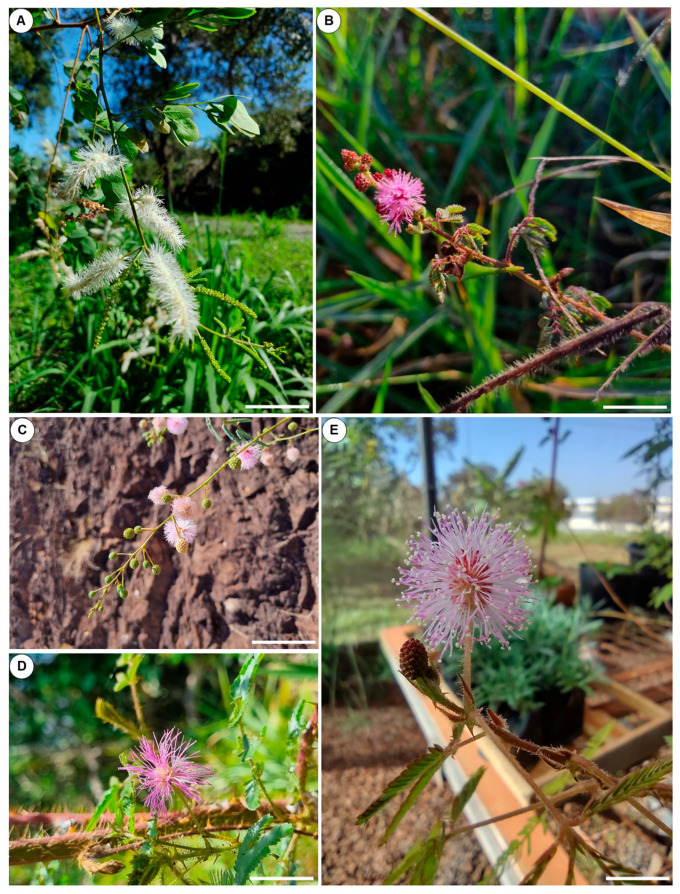
Flowering branches of the studied *Mimosa* species. (**A**): Flowers arranged in spikes with light-yellow staminal filaments and bipinnate leaves in *M. caesalpiniifolia*. (**B**): Flowers arranged in glomerules with pinkish filaments and bipinnate leaves in *M. diplotricha*. (**C**): Flowers arranged in glomerules with pinkish filaments in *M. myrioglandulosa*. (**D**): Flowers arranged in glomerules with pinkish filaments and bipinnate leaves in *M. paludosa*. (**E**): Flowers arranged in glomerules with pinkish filaments and bipinnate leaves in *M. pudica*. Scale: 2 cm. Images (**B**,**C**) by Ana Julia Peracini. All other images presented are by the authors.

Secretory cells were studied in developing and fully developed petals and leaf blades of *Mimosa caesalpiniifolia* (Figure 6A), *M. diplotricha* (Figure 6B), *M. myrioglandulosa* (Figure 6C), and *M. paludosa* (Figure 6D), and in sepals of *M. pudica* (Figure 6E) (Table 1), according to their distribution previously reported in [29]. To this end, vegetative and reproductive buds, leaves and flowers were collected in different municipalities in the state of São Paulo, Brazil (Table 1) between December 2022 and September 2023.

Anatomical analyses were performed on vegetative and reproductive buds fixed in neutral buffered formalin for 24 h, dehydrated through a graded ethanol series, embedded in histological resin [55], and longitudinally sectioned at 3–3.5 µm using a Leica RM2245 rotary microtome (Wetzlar, Germany). Sections were stained with 0.05% toluidine blue in phosphate buffer (pH 6.8) [56] for anatomical analysis and detection of mucilage (pink staining) and phenolics (green staining), mounted in water, and examined under light microscopy (LM). Images were captured using a Leica DFC295 digital camera (Wetzlar, Germany) coupled to a Leica DM5000 B light microscope (Wetzlar, Germany).

Ultrastructural analyses were performed on very small samples (0.5 × 0.5 cm) of sepals, petals, and leaf blades fixed in Karnovsky solution for 24 h [57], washed in 0.1 M phosphate buffer (pH 7.2) for 2 h at 4 °C, post-fixed in 1% osmium tetroxide, embedded in Araldite resin, and sectioned using a Leica EM UC7 ultramicrotome (Wetzlar, Germany). Semi-thin sections (0.5–1 µm) were stained with 0.05% toluidine blue in citrate buffer (pH 6.8) [56], mounted in water, and examined under light microscopy (LM). Ultrathin sections (60–70 nm) were contrasted with 2% uranyl acetate [58] and lead citrate for 15 min [59], and examined using a JEOL 100CXII transmission electron microscope (Tokyo, Japan). Images were captured using a Leica DFC295 digital camera (Wetzlar, Germany) coupled to a Leica DM5000 B light microscope (Wetzlar, Germany).

## 5. Conclusions

Our findings, together with previous anatomical data for *Mimosa* species [29], expand the current understanding of secretory cell biology in the genus. They reveal a pattern of dual-domain storage of distinct chemical compounds (mucilage and phenolics) within individual secretory cells. In contrast to other secretory systems, such as laticifers and nectaries, where diverse compounds are co-stored within a single compartment [1], the secretory cells described here exhibit clear spatial separation of metabolites into specialized intracellular domains. Notably, this study provides cellular ultrastructure data for five *Mimosa* species, representing a broad sample for transmission electron microscopy studies, thus strengthening the consistency of our observations within the genus.

## Figures and Tables

**Table 1:** Information on the studied *Mimosa* species and the sampled organs.

Species	Voucher	Organ	Locality of Collection
*M. caesalpiniifolia*	SPFR 17785	Sepal	Ribeirão Preto, Brazil
*M. diplotricha*	T. A. De Sousa 59 (SPFR)	Petal, leaf blade	Pirassununga, Brazil
*M. myrioglandulosa*	T. A. De Sousa 60 (SPFR)	Petal, leaf blade	Usina de Estreito, Pedregulho, Brazil
*M. paludosa*	T. A. De Sousa 61 (SPFR)	Petal, leaf blade	Usina de Estreito, Pedregulho, Brazil
*M. pudica*	T. A. De Sousa 58 (SPFR)	Petal, leaf blade	Ribeirão Preto, Brazil

## Data Availability

The original contributions presented in this study are included in the article. Further inquiries can be directed to the corresponding author.

## Abbreviations

The following abbreviations are used in this manuscript:

LM	light microscopy
TEM	transmission electron microscopy
